# Pain Control after Total Knee Arthroplasty: Comparing Intra-Articular Local Anesthetic Injection with Femoral Nerve Block

**DOI:** 10.1155/2015/649140

**Published:** 2015-05-03

**Authors:** Shengchin Kao, Hungchen Lee, Chihwen Cheng, Chingfeng Lin, Hsini Tsai

**Affiliations:** ^1^Department of Anesthesiology, Chang Gung Memorial Hospital, Linkou, Taoyuan City 333, Taiwan; ^2^Graduate Institute of Clinical Medical Sciences, Chang Gung University, Taoyuan City 333, Taiwan

## Abstract

*Background*. Direct intra-articular injection of low doses of local anesthetic (IALA) after closure of the joint capsule remains controversial for pain control after total knee arthroplasty (TKA). *Methods*. A retrospective study comparing patients receiving IALA with high doses (0.5% bupivacaine 60 mL) of local anesthetics or FNB in addition to intravenous patient-controlled analgesia with opioids for pain management after TKA was conducted. The primary end point was to compare the analgesic efficacy and early ambulation between the two groups. *Results*. No significant differences between the two groups in pain intensity, cumulative opioid consumption, incidences of opioid-related side effects, the time interval from the end of operation to the first time the patient could walk assisted with a walker postoperatively, and postoperative hospital stay were identified. Three patients in the IALA group but none in the FNB group walked within 12 hours after the end of operation. *Summary*. IALA with high doses of local anesthetics provides comparable analgesic efficacy as single-shot FNB after TKA and might be associated with earlier ambulation than FNB postoperatively.

## 1. Introduction

Postoperative pain management for total knee arthroplasty (TKA) is a challenge for clinicians. Inadequate pain relief after TKA may hinder early rehabilitation [1], delay discharge from hospital [2], and adversely affect functional outcomes [3]. Systemic opioid analgesia is limited by opioid-related side effects such as nausea, vomiting, dizziness, sedation, and respiratory depression. Epidural analgesia provides better pain relief than systemic opioids but is associated with more frequent urinary retention and hypotension after TKA [4]. In the last decade, femoral nerve block (FNB) as a part of multimodal analgesic regimens has been recommended as the technique of choice for postoperative pain management following TKA [5, 6] because FNB provides comparable analgesic effect to epidural analgesia but less side effects than those associated with systemic opioids or epidural analgesia [6, 7]. Nevertheless, FNB invariably results in femoral quadriceps muscle weakness, which may interfere with early ambulation after TKA [8] and is associated with an increased risk of falling [9]. In an effort to preserve quadriceps muscle power, an alternative analgesic technique consisting of peri- and intra-articular infiltration of a large volume of local anesthetics in the knee (LIA) has been developed as a part of multimodal analgesic regimens for TKA [10]. LIA has been shown to reduce pain intensity and opioid analgesics consumption after TKA when compared with systemic opioids alone [11, 12]. When compared with FNB, LIA produced similar analgesic efficacy at rest and less severe pain upon movement [13]. Another analgesic technique preserving femoral quadriceps muscle strength employs direct intra-articular injection of local anesthetics (IALA) after closure of the knee joint capsule. However, the analgesic efficacy of IALA for TKA is still controversial [14–18] and, to our knowledge, has not been compared with that of FNB.

At our hospital, the acute pain service team has been used to provide a single-shot FNB in combination with intravenous patient control analgesia (i.v. PCA) with opioids for postoperative pain management for TKA. Since 2010, some patients received IALA for pain management after TKA while others still received FNB. The aim of this study was to evaluate the analgesic efficacy of IALA by comparing IALA to FNB in terms of their pain intensity, opioid consumption, opioid-related side effects, and time to ambulation after TKA.

## 2. Materials and Methods

After approval from the institutional review board of Chang Gung Memorial Hospital (102-3855B), patients using i.v. PCA for postoperative pain control after knee surgeries between January 2010 and December 2011 were identified from the acute pain service database and their medical records were retrieved from hospital database. Only those patients who received unilateral primary TKA for knee osteoarthritis were included in the analysis. The exclusion criteria included age less than 18 years, use of opioids for more than 2 weeks prior to the surgery, operation under spinal anesthesia or peripheral nerve block, a history of alcohol or drug abuse, and use of dual therapy of IALA and FNB for postoperative pain management. Only patients who received the TKA under general anesthesia were included since the analgesic duration from spinal anesthesia and peripheral nerve block after operation can vary significantly. At our institution, the postoperative pain after TKA was managed with an ultrasound-guided, single-shot FNB with 0.2% levobupivacaine 25 mL in addition to i.v. PCA constituted with fentanyl on arrival to the postanesthetic care unit (PACU), and i.v. PCA was discontinued on the second postoperative day. Acetaminophen 500 mg 4 times a day and NSIADs were prescribed at the surgeons' discretion during hospital stay. At the PACU, additional opioids or ketorolac 30 mg i.v. could be administered if the intensity of pain was identified as equal to or more than moderate on a verbal severity scale (no pain, mild, moderate, severe, and extreme pain). All patients were given intravenous ondansetron 8 mg at the end of operation and were instructed to use i.v. PCA if his or her pain was equal to or more than moderate in severity. In the morning of first and second postoperative day (POD1 and POD2), the acute pain service personnel recorded the cumulative consumption of fentanyl from i.v. PCA, pain intensity, and the presence of opioids-related side effects including nausea, vomiting, dizziness, drowsiness, pruritus, and respiratory depression if any. The rehabilitation protocol began on POD1. Since 2010, some patients received 60 mL 0.5% bupivacaine injected into the knee joint (IALA) by the surgeon after closure of the joint capsule before wound closure. Enrolled patients were grouped into IALA group and FNB group, depending on the pain management modality facilitated. The initial search included 182 medical records, of which 88 were excluded based on the exclusion criteria, leaving 39 patients in the IALA group and 55 in the FNB group.

The demographic and clinical data recorded for analysis included gender, age, body weight, height, body mass index (BMI), preoperative comorbidities, American Society of Anesthesiologist physical status, history of alcohol or drug abuse or chronic opioid use, surgical approach, implanted prosthesis, intraoperative opioids, use of analgesics including ketorolac and opioids in the PACU, fentanyl consumption from i.v. PCA on POD1 and POD2, use of acetaminophen or NSAIDs in the ward, presence or absence of nausea, vomiting, dizziness, pruritus, and respiratory depression, and postoperative hospital stay. Pain intensity was assessed by verbal severity scale and recorded as 0–4 (0: no pain, 1: mild pain, 2: moderate pain, 3: severe pain, and 4: extreme pain). The amount of all opioids consumed in the PACU and via i.v. PCA was converted into i.v. morphine equivalence using the equianalgesic conversion ratios of meperidine : morphine = 75 : 10 and fentanyl : morphine = 0.1 : 10 [19, 20]. The cumulative POD1 and POD2 opioids consumption was calculated by adding the opioids administered in the PACU to those consumed via i.v. PCA recorded on the POD1 and POD2 morning, respectively. Comparisons were made between the IALA group and FNB group in regard to pain intensity, cumulative POD1 and POD2 opioid consumption, ketorolac injection in PACU, use of acetaminophen and NSAIDs in the ward, and incidences of nausea, vomiting, pruritus, dizziness, and respiratory depression.

To evaluate the impact of analgesic techniques on rehabilitation, the time interval from the end of operation to the first time the patient could walk assisted with a walker between the two groups was compared. The first time a patient could walk assisted with a walker after the operation was the most consistently documented landmark of rehabilitation progress in the medical records. A time interval less than 12 hours was considered as early ambulation, which was also compared between the two groups.

Statistical analysis was performed using the SPSS software package (version 17.0 for Windows, SPSS Inc., Chicago, IL, USA). Fisher's exact test was used to analyze the categorical data due to small expected counts, and 2-sample independent *t*-test was used to compare the continuous variables in the two groups. A two-tailed *P* value of less than 0.05 was considered significant.

## 3. Results

No significant difference in all demographic and preoperative medical conditions was noted between the IALA and FNB group except that more patients in the FNB group had a history of gastrointestinal diseases (gastritis, peptic ulcer, and gastroesophageal reflux disease) (*P* < 0.001) (Table 1). All patients in both groups received tricompartment prosthesis via medial parapatellar approach (Table 1). Slightly more opioids in morphine equianalgesic dose (mg/kg) were administered in the FNB group than in the IALA group during operation (0.29 ± 0.08 mg/kg versus 0.24 ± 0.05 mg/kg, *P* = 0.0018) (Table 1).

Postoperatively, there was no difference between the two groups regarding the use of ketorolac in the PACU and acetaminophen in the ward, but more patients in the FNB groups received NSAIDs in the ward (*P* < 0.0001) (Table 2). There was no statistical difference in pain intensity and cumulative opioid consumption per body weight (mg/kg) between the 2 groups on both POD1 and POD2, although a trend toward more total opioid consumption was noted in the IALA group than in the FNB group on POD2 (Table 3). No significant difference in the incidences of opioid-related side effects, the time interval from the end of operation to postoperatively first time walking assisted with a walker, and postoperative hospital stay was observed (Table 3). Three patients in the IALA group while none in the FNB group achieved early ambulation, but this difference is only marginally significant (*P* = 0.07) (Table 3).

## 4. Discussion 

Our analysis revealed that after TKA, IALA group and FNB group displayed similar pain intensity and cumulative opioid consumption in the first 2 days after operation. There were no significant differences in the incidences of opioid-related side effects, the time interval from the end of operation to first time walking assisted with a walker postoperatively, and postoperative hospital stay. A few patients in the IALA group walked within 12 hours after their operation while no patient in the FNB group did so. To our knowledge, this is the first report comparing the analgesic efficacy of IALA to that of FNB for pain management after TKA as well as their impacts on early ambulation. Our result suggested that IALA could be as effective as FNB in pain relief after TKA. Given that FNB is superior to i.v. PCA alone for pain reduction after TKA [7], IALA is supposed to be superior to i.v. PCA in a similar degree as FNB in the absence of the undesirable motor blockade.

Given the paramount importance of early rehabilitation and early ambulation after TKA, the ideal analgesic technique should provide adequate pain relief while preserving muscle strength. Therefore, LIA has been suggested as the preferred analgesic technique for TKA, considering its comparative analgesic effect to FNB in the absence of motor blockade [21]. IALA, on the other hand, has not been widely adopted for pain management after TKA as previous studies have yielded controversial results [14–18]. When compared with intra-articular saline injection, Ritter et al. [22], Browne et al. [14], and Rosen et al. [16] found no statistically significant difference in both pain score and opioid consumption in the first 24 hours after IALA. Badner et al. reported a decrease in opioid consumption in the first 24 hours after IALA without difference in pain score [15]. In Mauerhan's study [18] IALA reduced the pain score in the first 4 hours but there were no differences in opioid consumption as compared with placebo. Tanaka et al. showed that both pain score during the first 24 hours and opioid consumption in the first 48 hours were reduced after IALA [17]. The heterogeneity in study designs and settings may account for the discrepancy in results. The most prominent difference between these early reports and ours may be that the doses of local anesthetics used in these studies were much lower than that used in our study (Table 4). In our study, the doses of local anesthetic (60 mL 0.5% bupivacaine) used in IALA group were two- to sixfold than those used in these early reports. This high dose of local anesthetic in a large volume might conceivably contribute to the effective analgesia after TKA by IALA technique in our study. The plasma concentration of the similar doses of local anesthetics had been shown to be below the toxic level following LIA technique for pain control after TKA [23, 24] with their safety proved in numerous LIA studies [10, 11, 13, 21, 25, 26]. However, as the injection of local anesthetics was divided in 3 distinctive stages during the operation in LIA technique but accomplished in one shot in IALA, it remained unknown if the pharmacokinetics of local anesthetics in LIA could be extrapolated to IALA technique. Although no local anesthetic systemic toxicity was noted during our review of the medical records, it cannot be excluded that central nervous system intoxication during the postoperative period was mistakenly missed and treated as the residual effects of general anesthesia. As local anesthetic induced cardiovascular intoxication can be catastrophic, further researches are needed to delineate the pharmacokinetics of local anesthetics of IALA technique for TKA analgesia and to determine the optimal dosage of local anesthetics of IALA.

As the rehabilitation protocol in our hospital began on the POD1, time at which the motor blockade from a single-shot FNB with 25 mL 0.2% levobupivacaine had mostly dissipated, no difference was observed between the two groups in the time interval from the end of operation to postoperatively first time walking. However, albeit not statistically significant, a few patients in the IALA group but none in the FNB group could walk with a walker within 12 hours after operation, suggesting the superiority of IALA in preserving muscle power. To compare the impact of IALA on early ambulation to that of FNB, prospective studies incorporating immediate or shortly after operation rehabilitation protocols are needed.

Although the difference in intraoperative opioids administered was statistically significant between the two groups, its clinical significance was questionable. It seemed unlikely that this small difference, less than 2 mg in morphine equianalgesic dose, would exert significant impact on postoperative analgesia. Unexpectedly, postoperative use of NSAIDs in the ward between the two groups was statistically significant, which might be related to the preference of the attending physicians. Whether this difference contributed to the trend towards more cumulative opioid consumption in IALA group as well as its impact on pain relief and rehabilitation remained to be explored.

As with all retrospective studies, our analyses had a number of limitations, including post hoc selection of study variables, a lack of predetermined sample size, expectation bias, and missing data. The pain intensity was not rated by the visual analog scale or numeric rating score which might have revealed subtle but clinically significant difference in pain intensity. Our reviewed medical data did not record pain intensity during specific rehabilitative motion, which was especially relevant after TKA, even though the cumulative opioid consumption might reflect the intensity of pain during rehabilitation. Because the hospital discharge criteria were not standardized, the impact of different analgesic techniques on postoperative hospital stay could not be determined in current analysis. Additionally, as the use of NSAIDs was not standardized either, the extent of opioid-sparing effects was difficult to interpret. The reviewed medical records did not contain detailed information regarding the progress of rehabilitation; therefore the impact of analgesic techniques on rehabilitation could only be addressed by measuring the time interval from the end of operation to the first time walking assisted with a walker postoperatively. However, as ambulation was one of the most important components evaluated during rehabilitation after TKA, the time a patient was capable of walking assisted with a walker for the first time could be a valuable indicator of short-term outcome. There could be some time lag between the actual occurrence and the documentation of the time when a patient could walk with a walker for the first time after operation; nonetheless, this time lag was assumed to be comparable between groups. Finally, there was no consistent recording of functional recovery in the reviewed medical records; therefore, the impact of analgesic techniques on long-term functional outcomes could not be assessed.

## 5. Conclusion

Our retrospective analysis revealed that intra-articular injection of high dose local anesthetics provided similar analgesic efficacy as FNB and might be associated with early ambulation. Further studies are mandatory to confirm our findings in a prospective randomized controlled manner incorporating a rehabilitation protocol that is installed immediately or shortly after the operation to evaluate the impact of IALA on early ambulation after TKA. The pharmacokinetics of local anesthetics should also be determined before this practice can be widely recommended.

## Figures and Tables

**Table 1:** Comparison of demographic and clinical data between IALA and FNB group.

	IALA group (*N* = 39)	FNB group (*N* = 55)	*P* value
Gender (male/female)	9/30	7/48	0.266
Age (y), mean (SD)	68.41 (9.46)	70.64 (8.00)	0.221
BMI (kg/m^2^), mean (SD)	28.88 (7.12)	28.05 (3.88)	0.596
ASA physical status			0.076
I	3 (7.7)	0 (0)	
II	16 (41.0)	30 (54.5)	
III	20 (51.3)	25 (45.5)	
Comorbidities			
Pulmonary disease (%)	0 (0)	4 (7.3)	0.139
Cardiovascular disease (%)	22 (56.4)	38 (69.1)	0.267
Liver disease (%)	5 (12.8)	12 (21.8)	0.293
Renal disease (%)	1 (2.6)	0 (0)	0.415
Gastrointestinal disease (%)	10 (47.6)	47 (85.5)	<0.001
Surgical approach			
Medial parapatellar	39 (100)	55 (100)	N.A.
Implanted prosthesis			
Tricompartment	39 (100)	55 (100)	N.A.
Prosthesis manufacturer			
Zimmer	39 (100)	55 (100)	N.A.
Intraoperative opioids^∗^ (mg)	16.28 (3.58)	18.19 (4.92)	0.037
Intraoperative opioids^∗^/BW (mg/kg)	0.24 ± 0.05	0.29 ± 0.08	0.0018

Categorical data were analyzed using Fisher's exact test. Continuous data were analyzed using *t*-test.

BMI: body mass index.

BW: body weight.

ASA: American Society Anesthesiologist.

^∗^Converted into i.v. equivalent dose of morphine.

**Table 2:** Comparison of use of NSAIDs between IALA and FNB group.

	IALA group (*N* = 39)	FNB group (*N* = 55)	*P* value
Ketorolac 30 mg (i.v.) in PACU	9	15	0.81
Acetaminophen 500 mg QID in the ward	39	55	1.000
NSAIDs in the ward	18	54	<0.0001
Aceclofenac 100 mg BID	1	2	
Acemetacin 90 mg QD	0	1	
Acemetacin 90 mg BID	0	6	
Celecoxib 200 mg QD	0	29	
Celecoxib 200 mg BID	0	5	
Diclofenac 25 mg TID	0	1	
Diclofenac 25 mg QID	0	1	
Etodolac 400 mg QD	0	2	
Etoricoxib 120 mg QD	12	0	
Mefenamic acid 250 mg QID	0	4	
Naproxen 250 mg BID	0	1	
Parecoxib 40 mg Q12 H	5	2	

BID: twice daily; TID: thrice daily; QID: 4 times a day; Q12 H: every 12 hours.

**Table 3:** Postoperative pain intensity, cumulative opioid consumption, incidences of opioid-related side effects, time to ambulation, early ambulation, and hospital stay.

	IALA group (*N* = 39)	FNB group (*N* = 55)	*P* value
Verbal pain intensity scale^∗^			
POD1 (0/1/2/3/4)	0/34/4/1/0	0/51/3/1/0	0.622
POD2 (0/1/2/3/4)	0/37/1/0/0	0/48/0/0/0	0.442
Cumulative opioid consumption^∗∗^			
POD1 (mg), mean (SD)	38.21 (18.36)	35.55 (14.69)	0.437
POD2 (mg), mean (SD)	69.28 (33.43)	59.56 (29.40)	0.142
Cumulative opioid consumption^∗∗^/BW			
POD1 (mg/kg), mean (SD)	0.54 (0.23)	0.56 (0.26)	0.801
POD2 (mg/kg), mean (SD)	0.98 (0.43)	0.99 (0.48)	0.919
Opioid related side effects^∗∗∗^			
Dizziness (%)	5 (12.8)	6 (10.9)	1.000
Nausea (%)	2 (5.1)	3 (5.5)	1.000
Vomiting (%)	4 (10.3)	3 (5.5)	0.444
Time to walk (hrs), mean (SD)^∗∗∗∗^	33 (14)	35 (17)	0.68
Early ambulation (<12 hrs)	3	0	0.07
Postop hospital stay (day), mean (SD)	4.18 (0.91)	4.51 (1.41)	0.205

Categorical data were analyzed using Fisher's exact test. Continuous data were analyzed using *t*-test.

BW: body weight (kg).

POD1: postoperative day 1.

POD2: postoperative day 2.

^∗^Verbal pain intensity scale: 0 = no pain, 1 = mild pain, 2 = moderate pain, 3 = severe pain, and 4 = extreme pain.

^∗∗^Converted to i.v. equivalent dose of morphine.

^∗∗∗^No pruritus or respiratory depression was recorded in both groups.

^∗∗∗∗^The time interval from the end of operation to postoperatively first time walking assisted with a walker.

**Table 4:** Summary of previous studies on IALA.

Authors, years	Drugs and doses for IA injection (total volume)	Pain intensity	Opioid consumption
Badner et al., 1996 [15]	2 groups (30 mL) Saline (*n* = 27) 0.5% bupivacaine^∗^ (*n* = 27)	No difference in the first 24 h	↓ in the first 24 h
Mauerhan et al., 1997 [18]	4 groups (30 mL) Saline (*n* = 27) 5 mg morphine (*n* = 26) 50 mg bupivacaine (*n* = 24) 5 mg morphine + 50 mg bupivacaine (*n* = 28)	↓ in the first 4 h	No difference in the first 24 h
Ritter et al., 1999 [22]	4 groups (10 mL) Saline (*n* = 97) 0.25% bupivacaine (*n* = 114) 10 mg morphine (*n* = 109) 10 mg morphine + 0.25% bupivacaine (*n* = 117)	No difference in the first 24 h	No difference in the first 24 h
Tanaka et al., 2001 [17]	2 groups for OA knee (30 mL) 5 mg morphine + 0.25% bupivacaine^∗^ (*n* = 12) Saline^∗^ (*n* = 10)	↓ in the first 24 h	↓ in the first 48 h
Browne et al., 2004 [14]	2 groups (20 mL) Saline (*n* = 30) 0.5% bupivacaine^∗^ (*n* = 30)	Insignificant ↓ in the first 24 h (*P* = 0.07)	No difference in the first 24 h
Rosen et al., 2010 [16]	2 groups (100 mL) Saline (*n* = 24) 0.2% ropivacaine (*n* = 24)	No difference in the first 24 h	No difference in the first 24 h

^∗^With epinephrine 1 : 200,000.
